# Knowledge Graphs for drug repurposing: a review of databases and methods

**DOI:** 10.1093/bib/bbae461

**Published:** 2024-09-26

**Authors:** Pablo Perdomo-Quinteiro, Alberto Belmonte-Hernández

**Affiliations:** Grupo de Aplicación de Telecomunicaciones Visuales, Escuela Técnica Superior de Ingenieros de Telecomunicación, Universidad Politécnica de Madrid, Avenida Complutense 30, 28040 Madrid, Spain; Grupo de Aplicación de Telecomunicaciones Visuales, Escuela Técnica Superior de Ingenieros de Telecomunicación, Universidad Politécnica de Madrid, Avenida Complutense 30, 28040 Madrid, Spain

**Keywords:** drug repurposing, knowledge graphs, artificial intelligence, graph networks, explainability

## Abstract

Drug repurposing has emerged as a effective and efficient strategy to identify new treatments for a variety of diseases. One of the most effective approaches for discovering potential new drug candidates involves the utilization of Knowledge Graphs (KGs). This review comprehensively explores some of the most prominent KGs, detailing their structure, data sources, and how they facilitate the repurposing of drugs. In addition to KGs, this paper delves into various artificial intelligence techniques that enhance the process of drug repurposing. These methods not only accelerate the identification of viable drug candidates but also improve the precision of predictions by leveraging complex datasets and advanced algorithms. Furthermore, the importance of explainability in drug repurposing is emphasized. Explainability methods are crucial as they provide insights into the reasoning behind AI-generated predictions, thereby increasing the trustworthiness and transparency of the repurposing process. We will discuss several techniques that can be employed to validate these predictions, ensuring that they are both reliable and understandable.

## Introduction

Launching a new drug on the market is demanding, typically taking around 8.3 $\pm $ 2.8 years and costing $374.1 million [1]. This substantial investment has no guarantees, given the over 90% failure rate [2]. Including the cost of failures, the average cost rises to $1336 million [1]. The bottleneck in drug production is often in Phase 1, where approval rates range from 3.4 to 32.6% [1]. Since this phase tests drug safety and dosage, repurposing existing drugs, which have already been evaluated, is an alternative. Drug repurposing accelerates treatment discovery and minimizes failure risk, especially for rare diseases with fewer potential beneficiaries.

Drug repurposing is an attractive solution for pharmaceutical companies as it can quickly accelerate the finding of a treatment for a disease as well as minimize the risk of failure. This is specifically important in the context of rare diseases, where the small number of potential beneficiaries of the new treatment makes the investment less attractive. Notable examples of drug repurposing include sildenafil (Viagra), initially developed as an anti-hypertensive and later used for erectile dysfunction, generating significant revenue [3]. Semaglutide, originally for type II diabetes, is now also used for obesity and being studied for other diseases [4].

Traditionally driven by serendipity, drug repurposing now increasingly relies on computational approaches [5–7]. Among these, knowledge-based drug repurposing uses Knowledge Graphs (KGs), which organize information into nodes (entities) and edges (connections). Knowledge graphs integrate data from multiple sources, providing a comprehensive view. This review examines existing KGs in drug repurposing and their key details.

Various approaches can be employed to achieve drug repurposing using KGs. Typically, this problem is framed as a link prediction task, where the objective is to predict whether a particular drug node could potentially be connected to a disease node. Early research in this area began with systematic and data-driven methodologies, including protein–protein interaction network analysis [8, 9] and metabolic pathway studies [10], enabling a more structured exploration of potential drug repurposings.

Currently, the integration of KGs and artificial intelligence techniques has revolutionized the field of drug repurposing, offering a more sophisticated and efficient approach [11–16]. Knowledge graphs facilitate the organization and structuring of large volumes of biomedical data in a coherent manner, enabling the analysis of complex relationships among genes, diseases, drugs, and mechanisms of action. Artificial intelligence leverages this wealth of data to identify hidden patterns, generate hypotheses, and predict new indications for existing drugs with unprecedented accuracy and speed. However, it can be challenging to fully trust a prediction made by an AI model that lacks explanatory support. Nevertheless, the European General Data Protection Regulation states that

The data subject should have the right not to be subject to a decision, which may include a measure, evaluating personal aspects relating to him or her which is based solely on automated processing and which produces legal effects concerning him or her or similarly significantly affects him or her, such as automatic refusal of an online credit application or e-recruiting practices without any human intervention. ... In any case, such processing should be subject to suitable safeguards, which should include specific information to the data subject and the right to obtain human intervention, to express his or her point of view, to obtain an explanation of the decision reached after such assessment and to challenge the decision. [17] 

This is especially important in the health field, given that decisions made by AI can directly affect the health of individuals, so decisions must be carefully supported. In this context, the field known as eXplainable Artificial Intelligence (XAI) has emerged, aiming to provide explanations for the predictions made by black-box AI models. [18]. In this review,several XAI methods that are popular when trying to provide explanations to link predictions problems, and that could potentially be applied (or are already applied) in drug repurposing will be presented.

## KGs for drug repurposing

**Figure 1:**
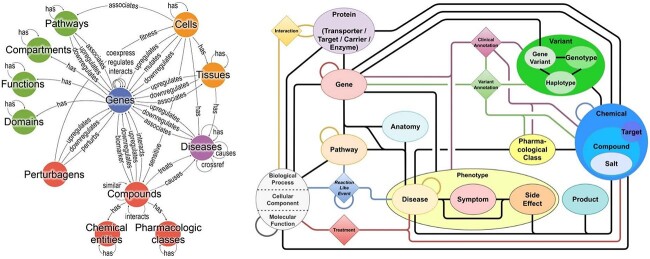
Two examples of existing KGs. Left: Bioteque KG [22] where 12 types of node and 67 types of edges are included. A total of 450 thousand biological entities and 30 million relationships are presented in the KG. Right: PharMeBINet KG [23] where 2 869 407 different nodes with 66 labels and 15,883,653 relationships with 208 edge types are included in this KG.

As stated above, KGs are data structures used to represent knowledge (Fig. 1). They usually represent knowledge of a specific domain, although we can also find more general KGs (i.e. Wikidata, DBpedia, or Freebase [19–21]). This structure is composed of nodes, that represent objects or entities; and edges or links, that represent connections between those entities. For instance, in a KG dedicated to drug repurposing, entities may include drugs, diseases, proteins, among others, while edges can denote relationships like ‘treats’, ‘interacts with’, or ‘causes’. In general, KGs can be undirected or directed. In the first case, it’s when the connections between nodes do not have a specific direction. In the case of drug repurposing, the connections usually have an established direction given the nature of the types of nodes that often present biological processes that start from one place and end in a consequence (for example, a drug can treat a disease, but a disease does not serve to treat a drug). When all nodes in the graph have only one and the same type, then this graph is called a homogeneous graph. A graph that has more than one node type and/or more than one edge type is called heterogeneous graph. A KG is a special type of directed heterogeneous graph that organizes information of a specific topic or domain. Many of them make use of semantics or ontologies to define their nodes and edges.

Various methods exist for representing knowledge in this manner. Typically, they start with a list of nodes alongside their respective attributes. These attributes can include diverse information, ranging from node descriptions to specific node properties (e.g. molecular weight for a represented molecule). On the other hand, edges can be depicted through different means. One approach involves an edge list, which enumerates the edges present within the KG. The space complexity of such representation is $\mathcal{O}(E)$, where $E$ is the number of edges in the graph. The edge list is really efficient in performing some basic operations like adding a new edge or vertex (time complexity of $\mathcal{O}(1)$) but it can be inefficient in some other operations such as finding if an edge exists between two nodes or checking the number of neighbors of a node (time complexity of $\mathcal{O}(E)$, where $E$ is the number of edges). Another commonly employed method is the adjacency matrix, a matrix of dimensions $N \times N$, where $N$ denotes the number of nodes in the KG, and $n_{ij} = 1$ signifies the presence of an edge between $N_{i}$ and $N_{j}$, while $0$ indicates otherwise. The space complexity for such representation is $\mathcal{O}(N^{2})$, where $N$ is the number of nodes in the graph; being usually less memory efficient that the edge list. However, this representation is really efficient at performing some more complex operation, such as finding where two nodes are connected in the graph (time complexity of $\mathcal{O}(1)$) or finding all the neighbors of a given node (time complexity of $\mathcal{O}(N)$, where $N$ is the number of nodes in the graph) As for data formats, KGs are typically available in tabular formats (.csv or.tsv) or structured data formats (RDF or JSON). Next, several KG will be examined that have been used (or have great potential) to tackle the drug repurposing problem. A summary of the information of each KG presented below can be found in Table 1.

**Table 1:** Table containing topological information about different biological KGs (number of nodes, number of edges, number of types of nodes, number of types of edges, size, and time of last update

**Name**	**Number of nodes**	**Number of edges**	Number of types of nodes	Number of types of edges	Size	Last Update
OREGANO [24]	88 937	824 231	12	19	250Mb	10 Nov 2023
CKG [25]	16 million	220 million	19	57	80Gb	17 Aug 2021
PrimeKG [26]	129 375	4 050 249	10	30	1Gb	25 Apr 2022
PharMeBINet [23]	2 869 407	15 883 653	66	208	1.8Gb (.zip)	5 Jan 2024
MSI [27]	29 959	478 728	4	5	539Mb (.zip)	19 Mar 2021
HetioNet [28]	47 031	2 250 197	11	24	12Mb (.sif.gz)	12 Apr 2016
DRKG [15]	97 238	5 874 261	13	17	383 Mb	4 May 2020
BioKG [29]	11 479 285	42 504 077	12	52	347 Mb	21 Mar 2024
KEGG [30]	–	–	5	10	Daily Updated	Daily Updated
DrugCombDB [31]	3011	6 891 566	2	1	761 Mb	31 May 2019
NeDRex [32]	275 759	2159159	6	8	509 Mb	25 Nov 2021

###  

#### 0.1 Bioteque

In Bioteque [22], information is structured into 12 types of biological entities such as genes, diseases, tissues, cells, etc. For each of these entities, it includes a range of descriptors or characteristics, such as the mutation pattern of a gene, the profile of physical interactions of the resulting proteins, the gene’s expression in different cell types, or its relationship with diseases. Among the 12 biological entities, the system encompasses around 1,000 types of descriptors. The data, sourced from 150 databases, were standardized and converted into numerical descriptors for algorithmic interpretation, facilitating the computational exploration of biological networks and connections.

###  

#### 0.2 OREGANO

OREGANO [24], a KG tailored for drug repurposing, distinguishes itself by encompassing a wide array of node types, including drugs, genes, phenotypes, diseases, and notably, natural compounds. This incorporation of natural compounds holds particular significance, especially with advancements in analytical tools facilitating deeper exploration of these compounds for disease treatment [33]. Drawing from approximately 10 curated databases and ontologies such as DrugBank, Uniprot, REACTOME, and HPO, this database includes a substantial compilation of information. In its entirety, OREGANO contains 88 937 nodes across 12 distinct node types and 824 231 edges with 19 edge categories.

###  

#### 0.3 Clinical Knowledge Graph

The Clinical Knowledge Graph (CKG) [25] stands out as one of the most expansive KG within the drug repurposing domain. This colossal KG includes data from 26 biomedical databases and 9 ontologies, culminating in a rich repository of knowledge. With a staggering 16 million nodes and over 220 million relationships, CKG represents a vast expanse of biomedical information. Its nodes are classified into 19 distinct types, including publications, drugs, diseases, proteins, and clinical variables, while its edges are categorized into 57 types, facilitating intricate relationship representation. Also, CKG offers various algorithms and machine learning techniques that can be used for data analysis.

###  

#### 0.4 PrimeKG

PrimeKG [26] emerges as a KG seamlessly integrating data from 20 diverse resources, biorepositories, and ontologies. With approximately 129 375 nodes and 4 050 249 relationships, PrimeKG contains a substantial wealth of information. Within its framework, PrimeKG organizes nodes into 10 distinct types, including Biological Process, Protein, Disease, Phenotype, Anatomy, Molecular function, Drug, Cellular Component, Pathway, and Exposure, facilitating comprehensive knowledge representation. Moreover, PrimeKG features 30 types of relationships, providing a detailed understanding of connections between various entities within the KG.

###  

#### 0.5 HetioNet

HetioNet [28] stands as another KG primed for drug repurposing, aggregating data from 29 distinct public resources. With 47 031 nodes and 2 250 197 relationships, HetioNet harbors a substantial repository of information. Comprising 11 diverse node types and 29 relationship types, it offers a comprehensive perspective on biomedical data. Accessible via their Neo4j server, HetioNet provides a user-friendly gateway to explore its wealth of interconnected information.

###  

#### 0.6 PharMeBINet

PharMeBINet [23] represents a significant evolution from HetioNet, building upon its foundation by integrating data from 19 additional public resources in addition to the original 29 sources. This expansion results in a noteworthy augmentation of nodes, increasing from 47 031 to 2 869 407—an impressive 61-fold increase. Similarly, the relationships within PharMeBINet experience a notable growth, escalating from 2 250 197 to 15 883 653—a sevenfold amplification. Moreover, PharMeBINet’s node diversity expands exponentially from 11 to 66 types, while the variety of edge types increases from 24 to 208. This transformative growth highlights PharMeBINet’s enhanced capacity to offer a more comprehensive and intricate representation of biomedical data.

###  

#### 0.7 Multiscale Interactome Network

The Multiscale Interactome Network [27] stands as a KG composed of drugs, diseases, human proteins, and biological functions. With 29 959 nodes and 478 728 relationships, it draws information from approximately 14 curated datasources, including DrugBank, The Human Reference Protein Interactome Mapping Project, and PsyGeNET. This KG provides a holistic perspective on the interplay between drugs, diseases, proteins, and biological functions, contributing valuable insights derived from a diverse set of curated data repositories.

###  

#### 0.8 DRKG

DRKG [15], a biological KG, includes 97 238 nodes and 5 874 261 edges. Constructed using data from six databases, including other KGs such as HetioNET, as well as bibliographical information, it offers a comprehensive repository of biological knowledge. With entities classified into 13 distinct node types and relationships delineated into 107 edge types, DRKG facilitates detailed exploration of biological interactions across various domains.

###  

#### 0.9 KEGG

The KEGG KG [30] is dynamically updated on a daily basis, making it challenging to know the precise count of nodes and relationships it encompasses. Drawing data from 18 databases, KEGG serves as a continuously evolving repository of biological knowledge. It contains a total of five node types and 10 edge types, facilitating the exploration of diverse biological interactions and pathways within its comprehensive framework.

###  

#### 0.10 BioKG

Another example of KG that can be used for drug repurposing is BioKG [29]. This KG is build using 18 different data sources, including DrugBank, UniProt, and KEGG. It contains a total of 7 node types (drugs, proteins, indications, diseases, gene ontology, expression, and pathways) and 10 edge types. One of the main advantages of this KG is that it contains a mapping module that allows it to be interoperable with other KGs.

###  

#### 0.11 DrugCombDB

DrugCombDb [31] diverges from traditional KGs designed for drug repurposing by focusing on identifying combinations of drugs rather than individual treatments for diseases. Unlike approaches aimed at determining which single drugs may be effective in treating a disease, DrugCombDb seeks to uncover synergistic combinations of drugs for therapeutic purposes. This specialized database includes 2887 drugs, 124 human cancer cell lines, and information regarding 448 555 unique drug combinations.

###  

#### 0.12 NedrexDB

NeDRexDB [32] is a graph database constructed by integrating data from 10 source databases through a crowdsourcing framework. It includes entities like proteins, genes, and drugs, as well as relationships between these entities. Each entity in NeDRexDB is assigned a unique identifier, facilitating integration across different data sources. The database addresses challenges such as inconsistent disease identifiers by using the Monarch Disease Ontology for diseases. NeDRexDB supports the exploration of biological networks for drug repurposing and disease module identification, leveraging the vast and interconnected data within. In total it contains 278 826 nodes and 2 327 974 edges, with a total of 6 node types (disorder, drug, gene, pathway, protein, signature) and 12 edge types. NeDRexDB is currently being updated into a second version that incorporates new node and edge types. At the moment of publication, NeDRexDB v2 contains 2 998 534 nodes and 10 803 818 edges.

## Problems to solve with KGs

Knowledge graphs offer a sophisticated method for organizing and analyzing complex, interconnected data, enabling a wide array of analytical tasks. Through semantic querying, pattern recognition, and inference, KGs can solve problems that require understanding intricate networks of connections within the data. From enhancing search engines to discovering new drugs, KGs provide a foundation for deriving insights, making predictions, and driving decisions across diverse domains.

One important application is link prediction, which involves estimating the probability of relationships between nodes based on the existing graph structure and attributes [34–36]. This process aims to identify potential edges not present in the graph by evaluating the likelihood of connections. Similarly, node prediction focuses on identifying and suggesting missing nodes within a KG based on existing connections and properties [37–39], estimating new nodes that fit well within the current graph structure.

Entity disambiguation is another critical task, which involves selecting the correct entity from a set of candidates using context and relational data [40, 41]. This is achieved by maximizing the likelihood function $L(e|C)$, where $e$ represents the true entity, given the context $C$. In the realm of graph-based classification, the goal is to categorize nodes or edges based on their features and structural properties [38, 42], assigning categories to nodes using a function $f:V \rightarrow Y$ that leverages the graph’s attributes and topology.

Community detection is another valuable application, involving the identification of subgraphs where nodes are more densely connected within clusters than between them [43, 44]. This process partitions the graph into subsets that maximize intra-cluster connections while minimizing inter-cluster connections. Additionally, pathfinding and relationship queries are essential for identifying the most relevant paths between entities [45, 46], finding sequences of vertices and edges that connect nodes based on criteria such as shortest distance or highest relationship strength.

Knowledge inference plays a crucial role in deriving new information or conclusions from existing data and relationships within the graph [47, 48]. Techniques like logical inference rules and probabilistic models are utilized to infer new connections. Anomaly detection focuses on identifying nodes or edges that deviate from expected patterns, highlighting unusual behaviors or properties [49]. This involves using statistical measures and machine learning models to detect anomalies.

Finally, graph embeddings transform the graph’s nodes, edges, and features into a low-dimensional space while preserving its structure [50–52]. This process facilitates the application of machine learning algorithms by simplifying the graph’s complex structure, making it easier to analyze and derive predictions from.

In the following section, we will cover different methods that will make use of KGs to obtain potential drug candidates for different diseases:

## Methods to solve KGs tasks

There are different methods that can be applied to analyze KG and make link predictions. Some of the most popular methods include the use of Deep Learning models (autoencoders and Graph Neural Networks, GNNs), random walks, translational embeddings, matrix factorization and metapath-based methods [53]. A summary of the performance, based on the metrics provided in the papers describing various AI methods, can be found in Table 2. Among the most popular method we find the Area Under the Receiver Operating Characteristic Curve (AUROC Curve) and the Average Precision (AP), often used to describe how well a classifier distinguishes two classes; and the Mean Reciprocal Rank (MRR) and Hits@k (where k is often 1, 3, 10, 50) which are evaluation metrics that describe how well a model has ranked a set of items. To obtain this latter metrics, a model is usually given a true label and a set of fake labels as input, the predictions are then ranked according to the score, expecting that the model will assign a higher rank (closer to 1) to the true prediction.

**Table 2:** Table containing different performance metrics on different KGs of the different AI model described throughout this review. The models are tested primarily through five different datasets: Cora [55], Citeseer [56], Pubmed [57], FB237 and WN18RR [58]. Among the metrics used to evaluate the models, we can find: Average Precision (AP), Area Under the Reciver-Operating Curve (AUC), Mean Reciprocal Rank (MRR), Hits@1, Hits@3 and Hits@10.

**Dataset**	Cora			Citeseer			Pubmed			FB237				**WN18RR**			
**Metric**	AP	AUC	MRR	AP	AUC	MRR	AP	AUC	MRR	MRR	Hits@1	Hits@3	Hits@10	MRR	Hits@1	Hits@3	Hits@10
GAE	96.35	95.91	28.98	98.55	98.27	63.33	96.36	96.37	16.67	–	–	–	–	–	–	–	–
NESS	98.57	98.13	–	99.5	99.43	–	96.52	96.67	–	–	–	–	–	–	–	–	–
GCN	–	95.01	35.5	–	95.89	50.01	–	98.69	19.94	–	–	–	–	–	–	–	–
GAT	–	93.9	31.86	–	96.25	48.69	–	98.2	18.63	–	–	–	–	–	–	–	–
SAGE	–	95.63	37.83	–	97.39	47.84	–	98.87	22.74	–	–	–	–	–	–	–	–
Node2Vec	–	90.97	37.29	–	94.46	44.33	–	93.14	34.61	–	–	–	–	–	–	–	–
NBFNet	–	92.85	37.69	–	91.06	38.17	–	98.34	44.37	41.5	32.1	45.4	59.9	55.1	49.7	57.3	66.6
MoCoSA	–	–	–	–	–	–	–	–	–	38.7	29.2	42.0	57.8	69.6	62.4	73.7	82.0
LMKE	–	–	–	–	–	–	–	–	–	40.4	32.4	43.9	55.6	69.6	62.4	73.7	82.0
TransE	–	–	–	–	–	–	–	–	–	27.9	19.8	37.6	44.1	24.3	4.3	44.1	53.2
DistMult	–	–	–	–	–	–	–	–	–	28.1	19.9	30.1	44.6	44.4	41.2	47.0	50.4
ComplEx	–	–	–	–	–	–	–	–	–	27.8	19.4	29.7	45.0	44.9	40.9	46.9	53.0
RotatE	–	–	–	–	–	–	–	–	–	33.8	24.1	37.5	53.3	47.6	42.8	49.2	52.6
AnyBURL	–	–	–	–	–	–	–	–	–	34.5	27.2	–	52.03	48.8	45.37	–	57.24
SAFRAN	–	–	–	–	–	–	–	–	–	38.9	29.8	–	53.7	50.02	45.9	–	57.8
Walkpool	–	95.9	–	–	95.94	–	–	98.72	–	–	–	–	–	–	–	–	–

###  

#### 0.13 Deep Learning Methods

##### 0.13.1 Graph autoencoder and variational graph autoencoder

Like other traditional deep learning methods, graph deep learning is based on neural networks. Some popular deep learning methods that have achieved good performance are autoencoders, where the idea is the same as traditional autoencoders, where a latent feature vector is obtained using and encoder, and then the original dataset is reconstructed using a decoder and the latent feature vector as input.

The two most known examples are GAEs (graph autoencoders) and VGAEs (variational graph autoencoders) [54]. This techniques make use of GNNs (Graph Convolutional Networks, GCNs) to create a latent feature matrix that is then used to reconstruct the adjacency matrix. For the learning of the VGAE, the approach is similar ti the one followed in regular autoencoders: 


(1) \begin{align*}& L = \mathbb{E}_{q(\boldsymbol{Z}|\boldsymbol{X}, \boldsymbol{A})} [log p(\boldsymbol{A}|\boldsymbol{Z})] - KL[q(\boldsymbol{Z}|\boldsymbol{X}, \boldsymbol{A})||p(\boldsymbol{Z})]\end{align*}

Where the first term represents the reconstruction loss, this is, how well does the new graph resemble the original graph; and the second term represents the regularization loss, which is commonly found in generative models and tries to ensure that the latent space distribution of encoded data approximates a Gaussian Distribution. In the case of GAEs, the objective will be to obtain a latent space matrix such that 


(2) \begin{align*}& \hat{A} = \sigma(\boldsymbol{Z}\boldsymbol{Z}^{T})\end{align*}

where $Z$ is obtained using a GNN (GCN). Another more recent approach that make use of graph autoencoders is NESS (Node Embeddings from Static Subgraphs) [59]. The idea behind NESS is to divide the graph into smaller non-overlapping subgraphs that will pass through the same encoder and decoder. By applying this modification, the model is able to increase its performance with respect to regular GAE. One of its main limitations, however, is that it only works in a transductive setting, which can be relevant in a drug repurposing setting where new knowledge is constantly being introduced.

##### 0.13.2 Graph Neural Networks

Similarly, GNNs are an adaptation of regular neural networks but applied to graph structures. The idea is to create a neural network structure for each node, where in each layer, the node gathers information from its neighbors. This way, a 1-layer GNN will gather information from its direct neighbors, while a 2-layer GNN will look at the 2-hop neighborhood. In each layer, there are two processes taking place, a message passing process and an aggregation process. During the message passing process, information from each neighboring node is used as input in a regular NN; then, during the aggregation process, the resulting vectors from the message passing operation are combined. There are multiple aggregation operations that can be used, including summation, pooling and average aggregators.

One of the first GNNs to appear were GCNs [54]. GCNs make use of the approach described above by following the next function: 


(3) \begin{align*}& H^{l+1} = \sigma (D^{-\frac{1}{2}}AD^{-\frac{1}{2}}H^{l}W^{l})\end{align*}

Where $H^{l+1}$ corresponds to the activation of neurons on the layer $l+1$; $A$ is the adjacency matrix (in fact, it is the adjacency matrix plus the identity matrix, which will allow for self loops), $D^{-\frac{1}{2}}$ is the square root of the degree matrix, used to normalize the activation according to $\frac{1}{\sqrt{d_{i}}d_{j}}$; $H^{l}$ corresponds to the activation of the previous layer; $W^{l}$ corresponds to the weights of layer $l$; and $\sigma $ corresponds to the activation function (i.e. ReLU or Sigmoid function).

One of the drawbacks, however, of GCNs is that they work in a transductive setting, using a graph of a fixed size; which can be a major inconvenience if we are using a KG that is constantly being updated. In this context, GraphSAGE [60] appears as an alternative that solves that issue. GraphSAGE extends GCN by allowing neighbor sampling; this is, instead of applying the message passing process through every node, it only makes use of a subgroup of neighbors. Additionally, it also incorporates the possibility to work in minibatches. By applying minibatches the space and time complexity of the mode can be fixed to: $\mathcal{O}(\prod _{i = 1}^{K} S_{i})$, where $K$ is the number of layers of the GNN and $S_{i}$ corresponds to the number of neighbors sampled on that layer.

Finally, another popular method are Graph Attention Networks (GAT) [61]. The idea of GAT is that, instead of applying a normalization that only relies on the degree of the nodes like GCNs, it makes use of an attention mechanism that assigns different weights to the neighboring nodes based on their relevance to the target node. This attention mechanism allows GATs to dynamically adapt the importance of each neighbor during the aggregation process, enabling more flexible and context-aware information propagation within the graph.

##### 0.13.3 Large Language Models

Other interesting approaches to solve the link prediction problem involve the use of Large Language Models (LLMs). One of such examples is LMKE [62]. LMKE uses a Masked Language Model (MLM) to obtain the predictions. It receives a head and a relationship (as well as their descriptions) and tries to predict the tail. This method achieves one of the best performances on the on WN18RR dataset [63].

Another example of an LLM applied to link prediction in KGs would be MoCoSA [64]. This model combines a structural encoder, which focuses on the structure of the graph—i.e. a translational embedding model—and a description encoder, which focuses on the descriptive information of entities, i.e. an LLM. This method is the current state of the art for link prediction on the OpenBG500 [65] and the WN18RR datasets.

###  

#### 0.14 Random Walks

Another approach of analyzing a graph is by using random walks. During random walk methods, several paths are created by randomly traversing the graph. These paths are later used to create different embeddings for each node. Some examples of random walk approaches are Node2vec [66], Dreamwalk [67], and WalkPool [68].

##### 0.14.1 Node2Vec

Node2Vec [66] uses different parameters to perform random walks to regulate the preference of performing a BFS walk or a DFS walk. BFS tries to stay as close as possible to the previous node, while DFS tries to move away from the previous node. This is done with parameters p and q. During each step, with a probability proportional to 1/p the walk can return to the previous node; with a probability proportional to 1/q the walk will continue towards a farther node (this is, a node that is a node that has a shortest distance of 2 from the previous node); it will move to any remaining node with a probability proportional to 1. Low $P$-values will result in BFS walks, while low q values will result in DFS walks.

Once the walks are created, a skip-gram model is trained to obtain different embedding for each node. A skip-gram model consists of a neural network with just one hidden layer that receives as input a one-hot feature vector of a target node and is trained to return a vector of size $N$, where $N$ is the number of nodes in the graph, that contains the probabilities of finding each node in the neighborhood of the target node. This probabilities are obtain thought the random walks. Once the model is trained, the hidden layer is used as feature vector for each node. This is a similar approach to Word2Vec embeddings for different words [69]. Recently more sophisticated versions of Node2Vec have appeared. One of such is Edge2Vec [70], which essentially makes used of the same architecture as Node2Vec but also utilizes edge information to obtain the predictions. This feature can be really useful in biomedical KGs where we have heterogeneous information with multiple edge types.

##### 0.14.2 Dreamwalk

Dreamwalk [67] is an AI method that makes use random walks to generated embeddings for drugs and diseases, which are later used as input for an XGBoost classifier. What Dreamwalk authors argue that regular random walk approaches are not very effective in solving the drug repurposing problem because the PPI network (gene-gene network) in KGs is much larger than the drug–disease, drug–gene, and gene–disease networks. This way, when randowalking the algorithms tends to stay in the PPI network. For this reason Dreamwalk uses biased random walks that allows for teleportation to semantically similar nodes. This is, if a disease or drug is reached in a random walk, there is a possibility of teleport to a node randomly sampled from a similarity matrix Sdrug or Sdisease, where the similarity values are used as sampling distribution. The model was tested in three KGs: MSI, HetioNet, and KEGG, achieving state of the art performance in the drug-disease prediction problem. Additionally, DreamWalk was tested on two different case studies, Alzheimer Disease and breast cancer, showing promising results.

##### 0.14.3 WalkPool

WalkPool [68] uses a mixed approach combining both GNNs and random walks. It starts by creating two subgraphs around the target link that is trying to be predicted: one that contains the predicted link and one removing the predicted link. Next it generates a feature vector for each node. This is done by using GNNs. Using the resulting feature vectors to obtain different weights and random walks, a matrix containing the transition probabilities is created. This matrix is then used to obtain different node, link and graph features that are fed into an MLP classifier. WalkPool is the current state of the art method that achieves the best performance in the link prediction task in the PubMed dataset [71].

###  

#### 0.15 Translational Embeddings

Translational Embeddings try to project the nodes and relationships into a latent space that satisfy a certain geometric property. For example, TransE [63], one of the most popular methods, tries to create embeddings for nodes and relationships in such way that Eh + Er is equal to Et, where Eh is the embedding of the head node, Er is the Embedding of the relationship and Et is the embedding of the tail node. Similarly, other methods have been developed that satisfy different geometric properties, allowing for reciprocal relationships (A--r->B and B--r->A), one to many relationships and many to many relationships. Some of these examples are DisMult [72], RotatE [73], TransD [74], and TransH [75].

##### 0.15.1 NBFNet

NBFNet [76] is another method that combines different ideas. It starts by generating all possible simple paths between two target nodes. It then obtains an embedding (using an embedding method like DistMul, TransE, or RotatE) suing each path as input. Next, all embeddings are aggregated into a single embedding which is then used as input for an MLP that obtains the final prediction. This model achieves the best performance in the FB15k-237 dataset.

###  

#### 0.16 Metapath

Metapaths method make their predictions based on a set of rules that are extracted from the graph. Metapaths, which are essentially rules, refer to sequences of entity types that aim to establish patterns representing the connections between entities. In a biological network, an example of a metapath that describes the relationship between a drug and a disease could be: ’Drug$\rightarrow $has_target$\rightarrow $Gene$\rightarrow $causes$\rightarrow $Disease’; indicating that a drug can potentially treat a disease if it targets the gene that causes the diseases. One of the main advantages of these methods is that they can provide human understandable explanations that can support the predictions. Two examples of methods that make use of metapaths are Metapath2Vec [77], AnyBURL [78], and SAFRAN [79].

##### 0.16.1 Metapath2Vec

One of the first methods that took into account the heterogenicity of graph when doing predictions was Metapath2Vec [77]. Metapath2Vec could also fit in the random walk category as it using a similar approach as Node2Vec. However, in this case, instead of making use of a completely random walk, the user can define several metapaths that are considered important, which will serve to bias the walk. This way, the model is able to capture the heterogeneous information of the graph.

##### 0.16.2 AnyBURL

AnyBURL [78] works by obtaining several rules (metapaths) through iterative traversals of the graph using random paths. After generating a path, rules are extracted from that path. Next, a (confidence) score is given to each rule and if it surpasses a certain confidence threshold the rule is stored. In the end, different rules are obtained that can be used to perform link prediction. To select the best candidate, given a head node and a relationship, AnyBURL selects the node that satisfies a rule with the highest confidence. If there is a tie, the second rule with the highest confidence is checked.

##### 0.16.3 SAFRAN

SAFRAN [79] works as an improvement of AnyBURL. The main difference is that instead of making the predictions based on the rule with the highest score, it makes its predictions based on the combination of scores of different rules. AnyBURL argues that combining the scores is not efficient as there is no guarantee that the rules are independent. However, to solve this issue, SAFRAM groups similar rules together and then a score is obtained, considering the score of different rules. This aggregation is efficiently performed using a MinHash scheme. Once the rules have been grouped, the final score is computed using a combination of the maximum value of each cluster. SAFRAN is among the top 5 methods than achieves the highest MRR value in the FB15k-237 dataset.

In the following section, different algorithms will be explored that can be used to support or provide hypothesis that can be used to validate the predictions.

## eXplainable AI

XAI is a growing field that tries to provide explanations to predictions obtained with AI models. There are countless reasons that make XAI an important aspect to consider when designing an AI pipeline. The first reason that may come to mind is that it provides explanations that can increase trust in the predictions that are made. This is especially important in the health domain, where a decision made by an AI can have a significant impact on people’s lives. Additionally, it can help to uncover possible errors or biases in the model, allowing us to improve the AI method or consider changing the input data. From a legal perspective, as it was stated in the introduction, the EU mandates ’the right to explanations’, which aims to provide users with a justification when an AI model is involved in a decision that concerns them. Finally, another crucial reason is that it is an invaluable tool in knowledge discovery, allowing us to understand complex patterns, trends, and insights hidden within large datasets. In this review, we will focus on several XAI methods that can be use in link prediction/drug repurposing.

###  

#### 0.17 GNNExplainer

GNNExplainer [80] is one of the first XAI methods applied to graphs, leveraging Mutual Information (MI) from information theory. MI quantifies the shared information between two random variables, measuring their dependence: 


(4) \begin{align*}& MI(Y, (G_{s}, X_{s})) = H(Y) - H(Y|(G = G_{s}, X = X_{s}))\end{align*}

**Figure 2:**
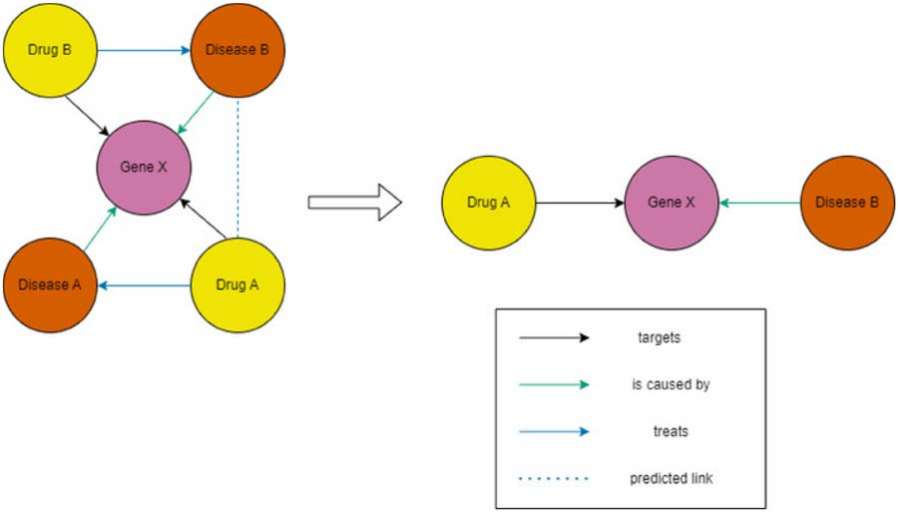
Example of an explanation produced as a subgraph. In this example, our AI model has predicted that Drug A could potentially be used to treat Disease B. A possible subgraph explanation could be that Drug A targets Gene X, which is one the genes that causes Disease B.

Here, $H(Y)$ is the entropy of the original predictions, and $H(Y|(G = G_{s}, X = X_{s}))$ is the entropy of the predictions using the subgraph. GNNExplainer aims to maximize the MI between predictions from the complete graph and a subgraph. Since $H(Y)$ is fixed, this is equivalent to minimizing $H(Y|(G = G_{s}, X = X_{s}))$. The optimization is achieved by training a mask on the adjacency matrix, with values between 0 and 1. This mask, when applied, weakens certain edges, effectively excluding them from predictions. The result is a subgraph that serves as the explanation (Fig. 2). A key issue is that retraining the mask for each explanation can lead to different results for the same instance upon repeated runs.

###  

#### 0.18 PGExplainer

PGExplainer [81] argues that directly maximizing MI can be very inefficient as it suggests iterating through every possible subgraph, with a complexity of 2^**^M. This way, it proposes a relaxation under the assumption that the subgraph (explanation) is a Gilbert random graph; this is, a random graph that is constructed where each edge has a probability of existing of P($e_{ij}$). This probabilities are obtained by using an MLP that receives as input embeddings produced by the GNN and produces as out the probabilities of generating each edge. The MLP is the trained trying to minimize H(Yo, Ys) (in other words, trying to maximize MI).

###  

#### 0.19 SubgraphX

SubgraphX [82] is a technique that utilizes Monte Carlo Tree Search (MCTS) and Shapley values. The former is often used in reinforcement learning algorithms, while the latter is a concept from game theory. The technique operates as follows: it starts with the entire graph as the root node, and then nodes are iteratively removed from the graph. In each iteration, several statistical values are computed, including the number of times a certain action is taken, the total reward, and the immediate reward.

###  

#### 0.20 DrugChat

Although not originally used for link prediction or drug repurposing, DrugChat [83] presents an intriguing approach that could be adapted for these tasks. DrugChat uses a Large Language Model (LLM) in a chatbot format to answer questions about drug compounds. The model takes a molecule represented as a SMILE graph and a prompt. The molecule graph passes through a pre-trained GNN to obtain an embedding. This embedding is then transformed into a format understandable by the LLM via an adapter. The LLM, also pre-trained, receives the prompt and the modified embedding to generate an answer. DrugChat is trained end-to-end with frozen weights for both the GNN and LLM; only the adapter’s parameters are trainable. The training data consists of Q-A pairs from curated databases like PubChem. This method allows for human-understandable explanations similar to ChatGPT. However, it shares common limitations with many LLMs, such as the risk of hallucinations, where the model provides convincing but incorrect answers.

###  

#### 0.21 PaGE-Link

PaGE-Link [84] is a method that also generates explanations as paths. It consists of two modules a k-core pruning module and a path-enforcing mask. The first module eliminates nodes with degree $< k$ to reduce complexity. Next, the path-enforcement module trains a mask similarly to the approach followed by GNNExplainer. However, this mask is trained using two loss terms: Lpred and Lpath. The former one is the term that tries to maximize MI (like GNNExplainer), while Lpath tries to select path-forming edges. This way, instead of obtaining a subgraph as explanation it obtains a path as explanation.

## Discussion and future directions

Knowledge graphs offer a sophisticated method to tackle the challenge of drug repurposing, showing significant variability in their structure and content. The Clinical Knowledge Graph (CKG) is the largest among those analyzed, with 16 million nodes and 220 million edges, while the Multiscale Interactome (MSI) graph is the smallest, containing 29 959 nodes and 478 728 edges. This variation highlights the diverse scales at which KGs can operate.

**Figure 3:**
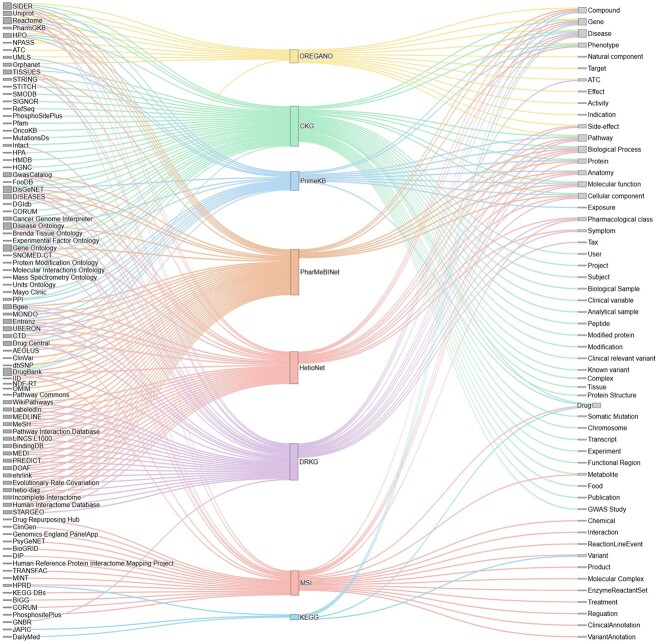
Overview of node types and data sources incorporated in various KGs as examined in this review. At the center of the illustration, the KGs are prominently displayed, illustrating their central role and connectivity within the data ecosystem. To the left, the existing data sources included in the studied KGs are listed, highlighting the diverse origins of the information that feeds into these graphs. On the right, the various node types included in the KGs are enumerated, demonstrating the range of entities and relationships modeled within these structures. This comprehensive depiction serves to underline the complexity and the multi-dimensional nature of the KGs, showcasing how they integrate disparate data types to foster enhanced analytical capabilities.

The number of entity and edge types also varies significantly across different KGs (Fig. 3). MSI has the fewest types (4 node types and 5 edge types), whereas PharMeBINet has the most (66 node types and 208 edge types). Despite these differences, certain entities like drugs, genes, and diseases are prevalent across nearly all graphs, which are essential for drug repurposing tasks. Some graphs might not explicitly label a node as a ‘drug’ but use terms like ‘compound’ or ‘treatment’ to represent similar concepts. Other common node types include proteins, anatomical structures, biological entities, molecular functions, and pathways.

The sources of information used to construct these graphs often overlap (Fig. 3). For instance, DrugBank is utilized in seven of the analyzed KGs, and DisGeNET, REACTOME, and SIDER are used in six. Furthermore, ontologies like Gene Ontology and Disease Ontology are frequently employed, providing structured data on genes and diseases. This overlap underscores the critical role these sources play in creating comprehensive and informative KGs.

Choosing the appropriate KG depends significantly on the intended predictive algorithm and the requirements for explainable AI (XAI). For instance, translational methods like TransE may not be ideal for frequently updated graphs like KEGG, as they require model retraining with each update. Conversely, for simpler explanation needs in the form of subgraphs with limited node types, smaller graphs like MSI or HetioNet might be more suitable. Computational resources are another critical consideration, as larger graphs demand substantial RAM, CPU, and GPU capacities. Therefore, selecting methods based on random walks, such as Node2vec or Dreamwalk, could be advantageous in resource-constrained environments compared to more demanding models like Large Language Models (LLMs) or GNNs.

Nonetheless, there are still some limitations that affect workflows that make use of KGs. One of such limitations is that often the knowledge depicted in KGs in incomplete, as collecting all the knowledge of a given area is often an X task. Additionally, long-term management of a KG is often costly, and therefore many KGs don’t contain updated information. This problem can be seen in column ’Last Update’ in Table X, where very few KGs contain information from the last 2 years. This issue often gives rise to predictions that do not offer new insights. Additionally, as described before, the size of many KGs can be a major drawback, as a high computational power may be required to work with them. Finally, one the limitations of drug repurposing approaches that make use of KGs is that they do not offer personalized medicine, but rather general potential drug candidates for diseases.

Evaluating AI models is challenging due to the diversity of metrics (AUC ROC, AUC PR, Hits@k, MRR, and MR) and the variety of KGs used for benchmarking (e.g. FB15k, FB15k-237, WN18, WN18RR, Cora, and Citeseer). This issue can be seen in Table 2 where the evaluation metrics and datasets selected by authors differ between methods. This variability complicates the assessment and comparison of models, making it difficult to identify a definitive ’best model’. Future work should focus on standardizing evaluation metrics and benchmarks to facilitate more consistent comparisons.

The type of output expected from AI models is also crucial. While most models predict drugs or diseases, others like DrugChat produce textual outputs, enabling the development of conversational agents. This interactive approach can enhance user engagement and understanding, particularly in clinical settings. As Large Language Models (LLMs) become more prominent, integrating such conversational interfaces could significantly improve the practical utility of drug repurposing tools.

When considering XAI approaches, the choice of AI method is pivotal. For example, if GNNExplainer is to be used, a GNN-based AI method should be selected. The desired type of explanation, such as subgraphs or metapaths provided by methods like GNNExplainer, PGExplainer, SubgraphX, and PaGE-Link, should guide the selection process. These methods offer human-readable explanations that are crucial for interpreting AI predictions.

In future research on AI applied to KGs, it is essential for the community to work towards establishing a consensus on performance evaluation. Currently, as illustrated in Table 2, researchers often rely on different KGs and diverse metrics to assess their methods, complicating the task of comparative analysis.

In conclusion, the interplay between KG characteristics, AI capabilities, and application context is complex. Future directions should aim at enhancing the interoperability of KGs, improving the scalability of AI methods, and developing more robust and explainable AI models. By addressing these challenges, we can better harness the potential of KGs for drug repurposing and other biomedical applications.

**Key Points**

- Conducted a comprehensive analysis of the unique features, commonalities, and differences of various Knowledge Graphs within the context of drug repurposing.
- Examined diverse AI methodologies applicable for identifying potential drug candidates.
- Investigated multiple Explainable AI (XAI) techniques to enhance the interpretability of the identified drug candidates.
- Suggested critical parameters to evaluate when selecting the appropriate Knowledge Graph, AI method, and XAI technique.
